# A pan-cancer analysis of EphA family gene expression and its association with prognosis, tumor microenvironment, and therapeutic targets

**DOI:** 10.3389/fonc.2024.1378087

**Published:** 2024-06-17

**Authors:** Zhe Cui, Chengwang Liu, Xuechao Wang, Yiping Xiang

**Affiliations:** ^1^ Division of Hematology and Transfusion Medicine, Tianjin Baodi Hospital, Tianjin Baodi Affiliated Hospital of Tianjin Medical University, Tianjin, China; ^2^ Department of Laboratory Medicine, Tianjin Baodi Affiliated Hospital of Tianjin Medical University, Tianjin, China; ^3^ Department of Pathology, Tianjin Medical University Cancer Institute & Hospital, National Clinical Research Center for Cancer, Tianjin's Clinical Research Center for Cancer, Key Laboratory of Cancer Prevention and Therapy, Tianjin, China

**Keywords:** Erythropoietin-producing human hepatocellular (Eph) receptors, pancancer, tumor microenvironment (TME), papillary renal cell carcinoma (KIRP), therapeutic targets, bioinformatics

## Abstract

**Background:**

Erythropoietin-producing human hepatocellular (Eph) receptors stand out as the most expansive group of receptor tyrosine kinases (RTKs). Accumulating evidence suggests that within this expansive family, the EphA subset is implicated in driving cancer cell progression, proliferation, invasion, and metastasis, making it a promising target for anticancer treatment. Nonetheless, the extent of EphA family involvement across diverse cancers, along with its intricate interplay with immunity and the tumor microenvironment (TME), remains to be fully illuminated.

**Methods:**

The relationships between EphA gene expression and patient survival, immunological subtypes, and TME characteristics were investigated based on The Cancer Genome Atlas (TCGA) database. The analyses employed various R packages.

**Results:**

A significant difference in expression was identified for most EphA genes when comparing cancer tissues and non-cancer tissues. These genes independently functioned as prognostic factors spanning multiple cancer types. Moreover, a significant correlation surfaced between EphA gene expression and immune subtypes, except for EphA5, EphA6, and EphA8. EphA3 independently influenced the prognosis of papillary renal cell carcinoma (KIRP). This particular gene exhibited links with immune infiltration subtypes and clinicopathologic parameters, holding promise as a valuable biomarker for predicting prognosis and responsiveness to immunotherapy in patients with KIRP.

**Conclusion:**

By meticulously scrutinizing the panorama of EphA genes in a spectrum of cancers, this study supplemented a complete map of the effect of EphA family in Pan-cancer and suggested that EphA family may be a potential target for cancer therapy.

## Introduction

Globally, the incidence and mortality of cancer are steadily rising on an annual basis (1). With the development of radiotherapy, chemotherapy, targeted therapy and immunotherapy, continuous endeavors are made to improve our comprehension of the intricate pathogenesis of tumors and elevate the standard of treatment (2). Nevertheless, further research is required to substantiate the efficacy of immunotherapy in various types of cancer (3). Pan-cancer analysis might help us unearth valuable factors in diagnosis, prognosis, and immunotherapy by analyzing genes in a wide variety of cancers and evaluating the similarities and variances in gene expression (4).

Erythropoietin-producing hepatocellular receptors (Ephs) constitute a significant subset within the realm of receptor tyrosine kinases (RTKs). Ephs can be classified into two distinct subfamilies, namely EphA and EphB, a differentiation primarily grounded in their structural attributes and the strength of their binding affinity with specific ligands known as ephrins. The EphA subfamily is comprised of nine individual members, namely EphA1, EphA2, EphA3, EphA4, EphA5, EphA6, EphA7, EphA8, and EphA10. These receptors assume critical roles not only in the regular progression of cell development but also in the advancement of various cancer types (5, 6), such as colorectal cancer (7), lung cancer (8), gastric cancer (9), hepatocellular carcinoma (10), and breast cancer (11).

The EphA family has long been identified as tumor neoantigens, regulating tumor cell stemness, invasion, and angiogenesis, and garnered considerable attention due to its potential as a target for anticancer therapies (12–14). Emerging evidence now also indicates they likely impact the tumor immune microenvironment, an area in which Eph receptors remain understudied (15). Considering that immune-checkpoint inhibitors have shown clinical success, albeit in a small percentage of patients, further research into EphA’s functions in controlling cancer immune-suppression is essential for comprehending and creating new targets against tumor immune evasion. So far there is no report on systematic analysis of EphA members from the perspective of pan-caner. Therefore, there is an evident need for performing pan-cancer analysis to achieve a comprehensive grasp of EphAs’ functionality and their role in tumor immune microenvironment. Such an understanding is essential to maximize the effectiveness of anticancer treatments that are directed toward EphA receptors.

In light of this, we provide a study about the complete spectrum of EphA’s activities and patterns of expression. The current study undertook a meticulous analysis of the expression profiles exhibited by all members of the EphA family across a diverse array of cancer types. This analysis leveraged data from TCGA databases to shed light on potential biological functions and shared characteristics of these receptors. Additionally, we delved into EphA’s impact on immune infiltration across a pan-cancer context, accompanied by an examination of individual cancer types.

## Materials and methods

### Data source

The RNA sequencing (RNA-seq) data (HTSeq-FPKM), along with corresponding clinical data and immune subtypes, were acquired from UCSC Xena (https://xena.ucsc.edu/, originated from TCGA database) (16).

### Expression analysis

The expression patterns of EphA genes in TCGA tumors were depicted through a boxplot graph. Subsequently, heatmaps were generated for 18 distinct tumor types, employing log2 (fold change) values to highlight discrepancies in EphA gene expression between primary tumors and adjacent normal tissues. Additionally, Spearman’s correlation test was employed to compute gene expression correlations among EphA members across 33 cancer types. To scrutinize the differential expression of EphA family genes across diverse cancer types, the “Wil-cox. test” was applied. For graphical representation, we utilized the “ggpubr” and “pheatmap” R packages to craft a box plot and a heatmap, respectively. The exploration of correlations within the EphA family genes involved the utilization of the “corrplot” R package.

### Survival analysis

We conducted univariate Cox regression and Kaplan-Meier (KM) analyses, facilitated by the “survminer” and “survival” R packages, to evaluate the influence of EphA on the survival outcomes associated with various cancers. Furthermore, the Cox proportional hazard model was applied to assess the connection between EphA gene expression and the prognosis of diverse cancer types. The “survival” and “forest plot” packages enabled the creation of a forest plot to visually present the results.

### Association of EphA expression with immune cell infiltration

We gauged the extent of immune and stromal cell infiltration across diverse cancers using immune scores and stromal scores metrics from ESTIMATE (17). The relationship between these scores and EphA expression was assessed using the Spearman Correlation Coefficient. Additionally, six distinct immune subtypes were delineated to quantify immune infiltration in the tumor microenvironment (TME) (18). Employing Analysis of Variance (ANOVA), we examined the link between immune infiltration types and SEMA3 expression within the TME based on immune subtypes acquired from TCGA pan-cancer data.

The Tumor Immune Estimation Resource(TIMER, https://cistrome.shinyapps.io/timer/) was developed for quantifying immune cell infiltration across 10,897 cancer samples from TCGA (19, 20). Leveraging TIMER gene modules, we analyzed EphA expression across diverse cancer types and explored its relationship with immune cell infiltration levels. Furthermore, correlation modules were utilized to examine the connections between EphA expression and gene biomarkers linked to tumor-infiltrating immune cells, utilizing established gene biomarkers (21, 22).

### Patients and tissues

KIRP tumor and adjacent normal kidney specimens were analyzed from a total of 157 patients with KIRP as part of a study approved by Tianjin Medical University Cancer Institute and Hospital. All patients were treated with radical or partial nephrectomy and rendered disease-free.

### Immunohistochemistry

Immunohistochemical staining was performed on the sections from surgical specimens fixed in 10% formalin and embedded in paraffin according to a standard method (23). Briefly, tissue sections were incubated with anti-EphA3 antibody (ab126261, Abcam; 1: 50 dilution) overnight at 4°C, and then incubated with secondary antibody (ab207995, Abcam; 1:100 dilution) followed by avidin-biotin peroxidase complex (DAKO) at room temperature for 30min. Finally, color development was performed with 3, 3′-diaminobenzidine. The immunostained slides were evaluated separately by two pathologists. The intensity of antibody staining was used to semiquantitatively quantify the expression of EphA3 in cancer cells. Staining intensity was categorized as follows: absent staining as 0, weak as 1, moderate as 2, and strong as 3.

### Statistical analysis

Statistical analyses were conducted using R 4.0.2 (https://www.r-project.org/). A linear mixed-effect model was employed to compare gene expression patterns between tumor and normal samples. Boxplots were utilized to illustrate gene expression variation across different cancer types. Univariate and multivariate Cox regression or Log-rank test were used to examine the relationship between gene expression and overall survival (OS) of patients. The correlation between gene expression and stemness scores, stromal scores, immune scores, and estimate scores was assessed using Spearman or Pearson correlation methods.

## Results

### Expression of EphA genes in pan-cancer

**Figure 1:**
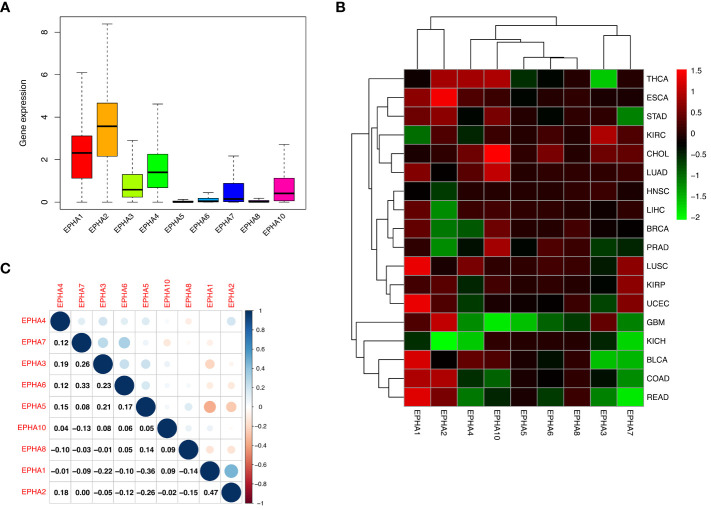
Our investigation delved into the expression of EphA genes across 33 cancer types utilizing the TCGA database. The outcomes unveiled a consistent up-regulation in the expression of EphA1, EphA2, EphA3, EphA4, EphA7, and EphA10 within cancerous tissues **(A)**. A deeper analysis highlighted intriguing dynamics, where specific EphA genes, namely EphA2 and EphA10 displayed overexpression in ESCA and CHOL, while EphA2 and EphA10 exhibited substantial down-regulation in KICH and GBM **(B)**. EphA1 and EphA2 emerged as the genes exhibiting the most robust positive correlation (Correlation coefficient =0.47). Conversely, EphA1 and EphA5 stood out as the two genes displaying the most prominent negative correlation (Correlation coefficient = -0.36, **C**).

The levels of EphA mRNA were assessed across 33 cancer types using data sourced from UCSC Xena, aiming to uncover the diversity inherent within the EphA family. The results revealed prominent high expression levels for most EphA genes in multiple cancer types. However, this trend was not mirrored by three genes: EphA5, EphA6, and EphA8, which exhibited relatively diminished expression levels (**Figure 1A**). When examining individual EphA genes like EphA2 and EphA10, significant up-regulation was observed in cases of esophageal carcinoma (ESCA) and cholangiocarcinoma (CHOL). Conversely, EphA2 and EphA10 displayed a down-regulation in kidney chromophobe (KICH) and glioblastoma multiforme (GBM) (**Figure 1B**). Particularly noteworthy was the correlation analysis that pinpointed the strongest pairwise correlation between EphA1 and EphA2 among the nine genes (Correlation coefficient =0.47). This finding implied potential shared characteristics or functions between these two genes. In contrast, EphA1 and EphA5 demonstrated a distinct negative correlation (Correlation coefficient =−0.36, **Figure 1C**), suggesting intricate co-expression interactions involving numerous EphA genes across diverse cancer types.

**Figure 2:**
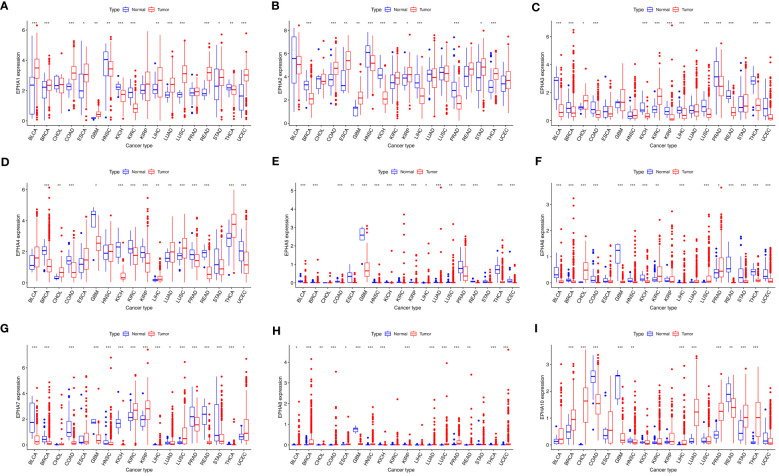
The visualizations derived from the TCGA database captured the expression patterns of EphA genes in diverse cancers. The EphA family genes include **(A)** EphA1; **(B)** EphA2; **(C)** EphA3; **(D)** EphA4; **(E)** EphA5; **(F)** EphA6; **(G)** EphA7; **(H)** EphA8; **(I)** EphA10. The outcomes consistently demonstrated distinct variations in the expression of EphA genes between cancerous tissues and their normal counterparts. *P < 0.05, **P < 0.01, ***P < 0.001.

On closer inspection, it became evident that nearly all EphA genes exhibited discernible disparities in expression between cancerous tissue samples and their normal counterparts (**Figure 2**). Moreover, significant differences in expression emerged across the spectrum of different cancer types.

### Prognostic value of EphA genes in pan-cancer

**Figure 3:**
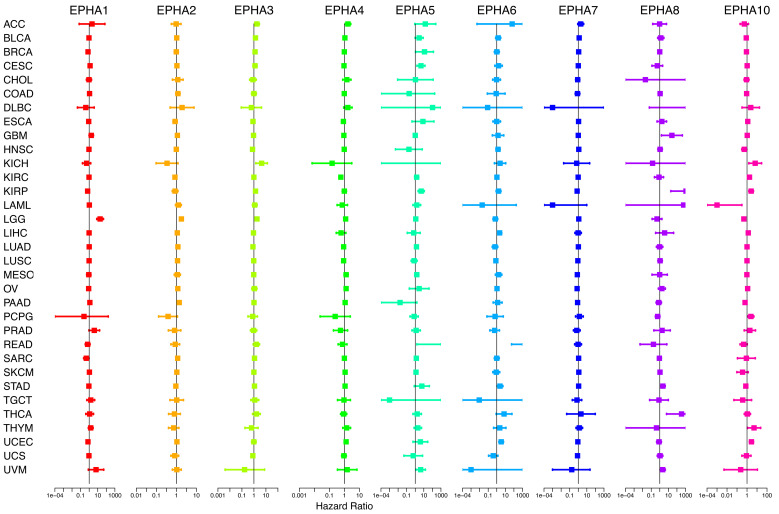
By applying COX analysis, we synthesized the prognosis risks associated with the nine genes across a pan-cancer context. The data underscored that EphA genes wielded the status of independent prognostic factors in various cancer types.

To deeply delve into the prognostic implications of EphA family genes, we examined the impact of the expression level of each gene on the prognosis of patients with specific cancers (**Supplementary Figure 1**). Through the application of the Cox proportional hazard model, the prognostic value of the nine EphA genes was evaluated across pan-cancer scenarios (**Figure 3**, **Supplementary Table 1**). The findings underscored a connection between EphA gene expression levels and the OS of patients, albeit with nuanced ramifications contingent on the specific cancer types. For example, heightened EphA5 expression correlated with an unfavorable prognosis of papillary renal cell carcinoma (KIRP) and uveal melanoma (UVM), whereas predicting improved survival in pancreatic adenocarcinoma (PAAD). Similarly, elevated EphA2 expression indicated a poor prognosis for patients with colon adenocarcinoma (COAD), diffuse large B-cell lymphoma (DLBC), low-grade glioma (LGG), and pancreatic adenocarcinoma (PAAD), yet correlated with higher survival rates in those with kidney chromophobe (KICH) and pheochromocytoma and paraganglioma (PCPG). Importantly, EphA genes emerged as independent prognostic markers for several distinct cancer types.

### Association of EphA genes with immune response and tumor microenvironment

**Figure 4:**
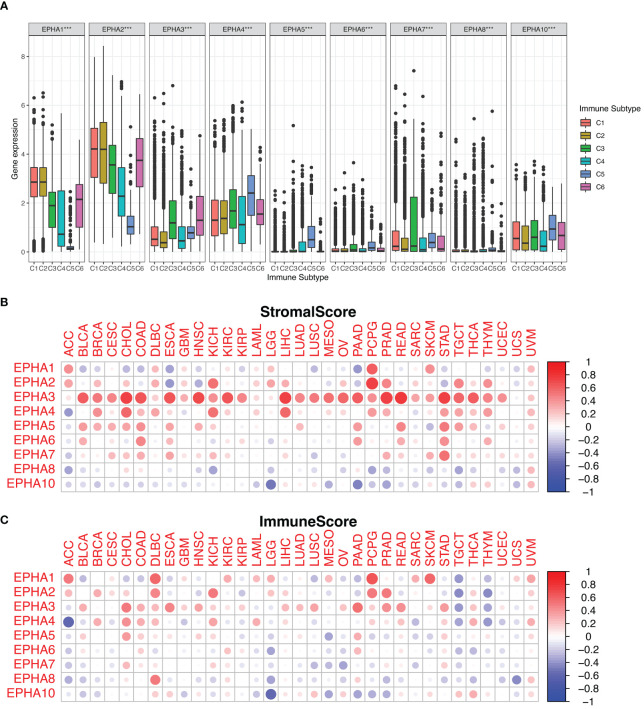
We investigated the correlation between EphA expression and immune infiltration across the pan-cancer landscape. The results depicted the significant roles played by all EphA genes within the TME **(A)**. Additionally, we harnessed the ESTIMATE algorithm to assess the stromal and immune scores attributed to EphA genes **(B, C)**. ***P < 0.001.

EphA genes occupy a critical position within the immune system due to their intricate involvement in the development, mobilization, and activation of both innate and adaptive immune cells (15, 24). Within the context of human malignancies, six distinct types of immune infiltration which were defined as the relative abundance of a set of immune cell populations, ranging from tumor promoting to tumor inhibiting, namely C1 (wound healing), C2 (INF-r dominant), C3 (inflammatory), C4 (lymphocyte depleted), C5 (immunologically quiet), and C6 (TGFβ dominant) (18). In the scope of our investigation, we conducted a thorough examination of immune infiltration patterns across the TCGA pan-cancer dataset, aligning them with the expression profiles of EphA genes (**Figure 4A**). Remarkably, the data illuminated connections between the expression levels of EphA genes and diverse categories of immune infiltration, except for EphA5, EphA6, and EphA8. Notably, EphA1 and EphA2 exhibited heightened expression in the C1 and C2 subtypes, indicating a plausible involvement in tumor promotion. This assumption found support in the observation of poorer survival rates among patients exhibiting these types of immune infiltration (C1 and C2). In contrast, EphA6 and EphA10 displayed elevated expression in the C3 and C5 subtypes, suggesting a potential tumor-suppressive effect. Nevertheless, it’s important to highlight that these correlations contradicted the roles of certain EphA members as promoters of cancer, which in some cases translated to diminished survival rates. A case in point was the poor prognosis linked to heightened EphA6 expression in UCEC (**Supplementary Figure 1**). These conflicting outcomes can potentially be attributed to the intricate biological diversity and multifaceted molecular interactions inherent to the progression of tumors. The implications of EphAs on the immune dynamics of the TME hold the promise of guiding novel pathways for the development of treatment strategies.

Furthermore, we delved into the stromal and immune scores indicative of tumor growth and metastatic potential. Leveraging the ESTIMATE algorithm, EphA genes were subjected to a comprehensive analysis involving these scores (**Figures 4B, C**). EphA genes with high stromal scores suggested heightened complexity within the TME, possibly intensifying tumor malignancy. An illustration of this was found in the close association between EphA3 expression and elevated stromal score across diverse cancers, whereas EphA10 expression demonstrated an inverse pattern. The associations between distinct gene expression levels within the EphA family and the estimated scores across various tumors underscored the diverse impacts that these genes might exert on the TME.

### Role of EphA genes in KIRP

**Figure 5:**
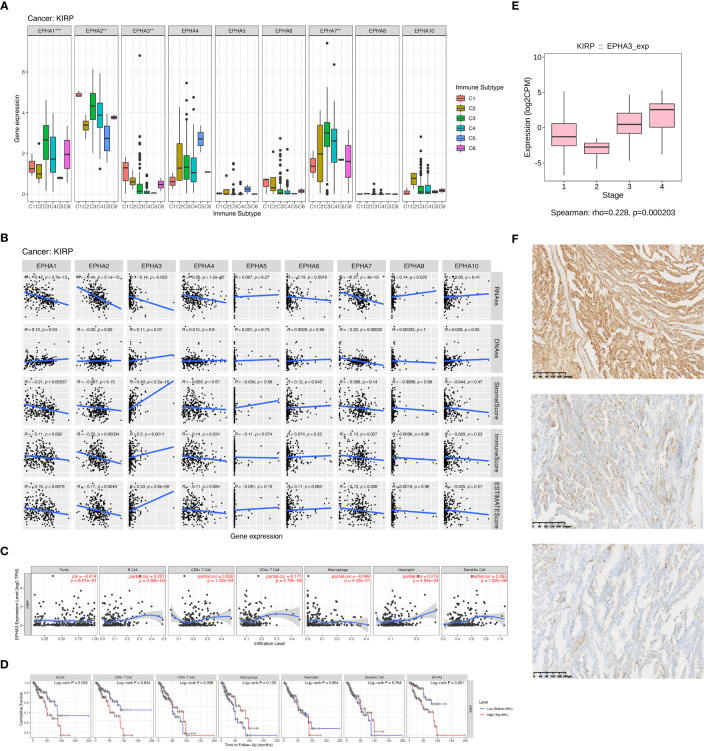
We delved into EphA’s impact on immune infiltration through an examination of individual cancer types. The outcomes of our investigation demonstrated significant differences in immune subtypes among EphA genes in KIRP **(A)**. Subsequently, we investigated immune infiltration in KIRP to evaluate the effects of these genes **(B)**. We also found that EphA3 expression displayed correlations with immune cell infiltration specific to KIRP **(C, D)**. Further, EphA3 was found to be up-regulated in advanced-stage KIRP samples analyzed by TCGA datasets **(E)**. Finally, we evaluated the correlation between EphA3 expression and clinicopatho-logical parameters in KIRP by immunohistochemistry **(F)**. The pictures showed the different degrees of staining of EphA3 in KIRP:(up) strong EphA3 expression in KIRP; (center) moderate EphA3 expression in KIRP; and (down) weak EphA3 expression in KIRP (Magnification x400). **P < 0.01, ***P < 0.001.

Renal carcinoma ranks as the 13^th^ most prevalent cancer worldwide, with an escalating incidence rate (25). Papillary renal cell carcinoma (KIRP), constituting 10% to 15% of kidney cancer cases, stands as the second most common subtype (26). In the metastatic context, the prognosis for KIRP patients remains bleak due to the absence of effective therapeutic options (27). The optimal treatment strategy for advanced KIRP continues to be a subject of debate. Nonetheless, recent clinical studies have unveiled promising outcomes for both molecularly targeted therapies and immunotherapy within this subtype (28). Given the elevated risk association of most EphA genes with poor survival in KIRP patients, our research expanded to explore the interplay between EphA genes, diverse immune subtypes, stem cells, and the TME in KIRP. The connection between EphA gene expression and distinct immune subtypes in KIRP echoed the patterns observed across all 33 TCGA cancer types. Of particular note, EphA1, EphA 2, EphA 3, and EphA 7 exhibited significant associations with immune infiltration profiles in KIRP (**Figure 5A**). Subsequently, we delved into the correlation between EphA expression and stromal score, revealing positive associations for EphA3 and EphA6 (P < 0.05) in KIRP, in which EphA3 demonstrated the most robust correlation (r =0.49) (**Figure 5B**). On the other hand, EphA2, EphA4, EphA5, EphA7 EphA 8, and EphA 10 did not display significant correlations with stromal scores, implying their potential origin from the tissue stroma in KIRP. Moreover, EphA3 exhibited correlations with the immune score, a metric assessing the presence of infiltrating immune cells (P = 0.0011) and tumor purity (Estimate score) (P < 0.0001).

Upon a deeper exploration of EphA3’s role, pronounced connections surfaced between EphA3 expression and various immune cell types, such as CD4^+^ T cells (R =0.171, P =5.79E-03), CD8^+^ T cells (R =0.236, P =1.32E-04), B cells (R =0.221, P =3.60E-04), dendritic cells (R =0.295, P =1.62E-06), and neutrophils (R =0.215, P =4.94E-04). However, these correlations were notably absent for tumor purity (R=-0.014, P=8.21E-01) and macrophages (R =-0.049, P =4.42E-01) (**Figure 5C**). Leveraging the TIMER database, the Kaplan-Meier curves highlighted a substantial linkage between KIRP patients survival and EphA3 expression (P =0.001), as well as the infiltration of CD8^+^ T cells (P =0.024) and B cells (P =0.035) (**Figure 5D**). Overall, our findings underscore the potential role of EphA3 in governing immune cell infiltration in KIRP. Alongside B cell and CD8^+^ T cell infiltration, EphA3 emerges as a pivotal modulator influencing clinical outcomes for KIRP patients.

Further delving into the connection between EphA3 expression and immune cell infiltration, we uncovered EphA3’s association with markers of various immune cell types, including B cells (CD19 and CD79A), monocytes (CD86 and CD115), M1 macrophages (INOS, IRF5, and COX2), Th2 cells (GATA3 and IL13), and Treg cells (FOXP3, CCR8, STAT5B, and TGFb) (**Table 1**). This revelation suggested that EphA3 may play a regulatory role as an immunomodulator in the renal cancer microenvironment.

**Table 1:** Correlations between EphA3 expression and related gene markers in KIRP.

Description	Gene markers	R	P
B cell	CD19	0.257	9.08E-06(***)
	CD79A	0.380	2.08E-11(***)
Monocyte	CD86	0.162	5.71E-03(**)
	CD115(CSF1R)	0.229	8.1E-05(***)
M1 Macrophage	INOS(NOS2)	0.253	1.35E-05(***)
	IRF5	-0.121	4.01E-02(*)
	COX2(PTGS2)	0.43	1.8E-14(***)
Th2	GATA3	0.447	1.14E-15(***)
	STAT6	0.056	3.42E-01
	STAT5A	0.095	1.07E-01
	IL13	0.157	7.48E-03(**)
Treg	FOXP3	0.279	1.37E-06(***)
	CCR8	0.336	4.27E-09(***)
	STAT5B	0.124	3.52E-02(*)
	TGFb(TGFB1)	0.36	2.51E-10(***)

*P < 0.05, **P < 0.01, ***P < 0.001.

KIRP, papillary renal cell cancer.

### The relationship between EphA3 protein expression and clinicopathologic parameters in KIRP

EphA3 was found to be up-regulated in advanced-stage KIRP samples analyzed by TCGA datasets (HR =0.228, P <0.001, **Figure 5E**). To further elucidate the association between EphA3 expression and KIRP, differences in the level of EphA3 protein expression between cancer cells and adjacent normal cells were compared in KIRP tissue specimens by immunohistochemistry. The high and low groups were defined based on the median EphA3 expression (**Figure 5F**). The result showed that the expression of EphA3 protein was significantly associated with metastases (TNM) stage (P =0.0017), tumor diameter (P <0.0001), and age (P =0.0030). No significant association between the expression of EphA3 and sex (P =0.8635) was found (**Table 2**).

**Table 2:** Expression of EphA3 protein in KIRP and the association with clinicopathologic parameters.

Parameter	Low expression (n=72)	High expression (n=65)	p-value
TNM Stage, n (%)			
I and II	64 (46.7%)	43 (31.4%)	0.0017
III and IV	8 (5.8%)	22 (16.1%)	
Sex, n (%)			
Female	33 (24.1%)	28 (20.4%)	0.8635
Male	39 (28.5%)	37 (27.0%)	
Age (years), n (%)			
<60	21 (15.3%)	36 (26.3%)	0.0030
≥60	51 (37.2%)	29 (21.2%)	
Tumor Diameter, cm			
<7	44 (32.1%)	16 (11.7%)	<0.0001
≥7	28 (20.4%)	49 (35.8%)	

## Discussion

This study offered an extensive pan-cancer analysis of EphA genes spanning 33 distinct cancer types using independent datasets from TCGA. The results emphasize the marked elevation in expression for the majority of EphA genes among cancer patients, except for EphA5, EphA6, and EphA8. Further, a thorough investigation into the association between the OS of patients and EphA expression levels was conducted. It was revealed that most of these genes hold prognostic significance within varied cancer types, often exerting bidirectional effects. EphA2, for example, correlated with a poor prognosis in COAD, DLBC, LGG, or PAAD cases, while heralding improved survival for patients with KICH and PCPG.

The TME stands as a pivotal determinant in tumorigenesis and tumor growth, offering a conducive setting for tumor proliferation and the dampening of immune responses (29, 30). Ephs and their corresponding ephrin ligands orchestrate intricate cell interactions during cellular growth processes, extending their influence to malignancies and the TME, thereby promoting cancer invasion, metastasis, and angiogenesis (24, 31). Prior studies have investigated six distinct types of immune infiltration (C1–C6) that may impact the proliferation of tumor cells in cancer patients (18). Three scenarios of immune infiltration integrated, respectively, by poor cytotoxicity(C1–C2), intermediate cytotoxicity (C3–C4), and high cytotoxicity(C5–C6). Tumors with highly cytotoxic immunophenotype would be partially repressed by the immune system, resulting in less frequent progression to more advanced stages. Our findings consistently point towards robust connections between most members of the EphA gene family and infiltration within the TME. Particularly notable are the correlations of EphA1 and EphA2 with more aggressive subtypes of immune infiltration subtypes (C1 and C2), indicative of a poorer prognosis. Employing the ESTIMATE method, we further unearthed associations between EphA genes and infiltration of stromal and immune cells. This observation aligns with previous studies highlighting the role of EphA as an immunomodulator and pro-inflammatory factor (24, 32–34). These genes, thus, emerge as prospective candidates for treatment targets or predictive markers for the effectiveness of immune checkpoint modulators in cancer patients.

EphA’s role exhibits divergence, potentially acting as either tumor-suppressive or tumor-promoting within the same tumor origin. For instance, the correlation pattern of these genes with immune infiltration subtypes in KIRP presented a parallel trend, with EphA3 demonstrating elevated expression in C1 and C2, thus implying a tumor-promoting function. Intriguingly, EphA3 demonstrated a robust correlation with stromal scores (r =0.49) in KIRP. Though these findings imply EphA3 is a promising target for anticancer treatment, further experimental validation is necessitated.

Elevated EphA3 expression is linked to poor prognosis in several malignancies including gastric cancer (35), colorectal cancer (36), and hepatocellular carcinoma (37). The study by Wang et al. suggested EphA3’s tumor-suppressive role in kidney renal clear cell carcinoma (KIRC) (38). To date, there has been little published research investigating EphA3’s role in the prognosis or therapeutic potential of KIRP. In our study, differential gene expression analysis accentuated significant down-regulation of EphA3 in KIRP samples compared to normal kidney samples (**Figure 2**). However, heightened EphA3 expression emerges as a risk factor for a poor prognosis in KIRP patients (**Figure 3**). This conflicting result adds complexity to our comprehension of EphA3’s contribution to KIRP initiation and development. To deepen our under-standing, we analyzed the correlation between EphA3 and clinical stages using the sequencing data and clinical information from the TCGA database. The results reveal that EphA3 was up-regulated in advanced-stage KIRP samples (**Figure 5E**). Notably, further analysis of clinical samples showed that overexpression of EphA3 was associated with tumor diameter (P <0.0001) and TNM stage (P =0.0017) (**Table 2**).

EphA3 is the first receptor with dual significance, being recognized as a tumor antigen in lymphoblastic leukemia cells and, independently, in melanoma cells from a patient with an EphA3-reactive T cell immunological response (39). Recently, advancements have further illuminated the role of EphA3 as a binding partner to PD-L1, discovered during an extensive search for transmembrane receptors engaged with immunoglobulin superfamily members. The connection between EphA3/PD-L1 co-expression and overall PD-L1 expression is intricately linked to a CD8 T effector cell signature in urothelial carcinoma tissues, as unveiled through gene expression analyses (31).To delve deeper into the significance of EphA3 in KIRP, an exploration was undertaken using the TIMER databases to uncover its correlations with B cells, CD8^+^ T cells, CD4^+^ T cells, and neutrophil infiltration. The results established the affiliation of EphA3 expression with markers of B cells (CD19 and CD79A), monocytes (CD86 and CD115), M1 macrophages (INOS, IRF5, and COX2), Th2 cells (GATA3 and IL13), and Treg cells (FOXP3, CCR8, STAT5B, and TGFb) (**Table 1**). These insights further accentuate the alignment of EphA3 expression with immune infiltration in KIRP, validating its role as a modulator of immune evasion within the renal cancer microenvironment.

In summary, our findings cast light on the multifaceted contributions of EphA to immune response and the complex landscape of the TME, which is essential for promoting personalized anticancer treatments. A groundbreaking assertion is made that elevated EphA3 expression independently heightens the risk of poor prognosis in KIRP patients, while concurrently functioning as a regulator of the immune microenvironment as well as a viable biomarker for prognostic evaluation and the assessment of immunotherapy response in patients with kidney cancer.

It’s imperative to acknowledge that the study carries certain limitations. First of all, the analysis of EphA genes leaned heavily on bioinformatics perspectives, lacking the validation provided by *in-vivo* or *in-vitro* experiments. More studies focusing on molecular and cellular basis are needed to facilitate high-throughput data analysis. Additionally, the inclusion of extensive data from diverse databases is essential to mitigate any potential information bias. Prospective studies investigating EphA gene expression in the context of immune cells across a wide range of cancers hold the potential to unveil novel insights, thereby opening new avenues for exploration in this intriguing domain.

## Conclusion

In theory, it may be possible to enhance tumorimmune therapy by modifying EphA activity. However, how EphA controls tumor immunity is still largely a mystery. On the one hand, a large number of research papers evaluating the role of Eph receptors in immune responses have not yet been applied to cancer models. On the other hand, EphA receptor kinase inhibitors have been developed, but it remains unclear how they effect on the immune system. In summary, despite a great deal of research on Eph receptors in immunology and cancer biology, this family stands mostly understudied in the context of tumor immunity. Here, we delve deeply into the multifarious roles played by EphA genes play in the initiation and progression of diverse cancers, alongside their associations with patient prognosis and immune response. This gap in our current knowledge identifies a distinct opportunity for new discoveries that may advance our understanding of the tumor microenvironment and pave the way for novel immunotherapeutic targets.

## Data availability statement

The datasets presented in this study can be found in online repositories. The names of the repository/repositories and accession number(s) can be found in the article/**Supplementary Material**.

## Author contributions

ZC: Conceptualization, Investigation, Software, Writing – review & editing, Writing – original draft. CL: Writing – review & editing, Conceptualization, Data curation, Methodology, Supervision. XW: Formal analysis, Project administration, Resources, Validation, Visualization, Writing – original draft. YX: Resources, Writing – original draft, Funding acquisition, Writing – review & editing.
