# Supplementary material for: A pan-cancer analysis of EphA family gene expression and its association with prognosis, tumor microenvironment, and therapeutic targets
Source: Front Oncol. 2024 Jun 17;14:1378087. doi: 10.3389/fonc.2024.1378087 (PMC11215048; doi:10.3389/fonc.2024.1378087)
Supplement: Supplementary file 1 [file DataSheet_1.docx]

**Supplementary Table 1:** The prognosis risk of EphA genes in pan-cancer by COX analysis

| **Gene** | **Cancer** | **HR** | **HR.95L** | **HR.95H** | **p-value** |
| --- | --- | --- | --- | --- | --- |
|  | LGG | 18.88773524 | 7.747174462 | 46.04860059 | 1.03E-10 |
|  | KIRP | 0.661272008 | 0.502208291 | 0.870715748 | 0.003218038 |
| **EphA1** | SARC | 0.46493726 | 0.227989357 | 0.948143626 | 0.03516769 |
|  | UCEC | 0.726150001 | 0.53818947 | 0.979754998 | 0.036278811 |
|  | BRCA | 0.733980756 | 0.544492676 | 0.989412299 | 0.042374442 |
|  | LGG | 1.776197389 | 1.493814666 | 2.111960229 | 7.87E-11 |
|  | PAAD | 1.38608308 | 1.17463045 | 1.635600631 | 0.000110743 |
| **EphA2** | LUAD | 1.189695938 | 1.044992446 | 1.354436992 | 0.008662919 |
|  | OV | 1.173645587 | 1.020021491 | 1.350406807 | 0.02529191 |
|  | KIRC | 0.847293057 | 0.719565385 | 0.997693246 | 0.046848717 |
|  | LGG | 1.799821426 | 1.3283732 | 2.438589673 | 0.000149255 |
|  | BLCA | 1.350212159 | 1.111113402 | 1.640762204 | 0.002531744 |
|  | KICH | 4.383849655 | 1.455441267 | 13.20433757 | 0.008611691 |
| **EphA3** | KIRP | 1.358696345 | 1.065974177 | 1.731801575 | 0.013284651 |
|  | BRCA | 1.244040509 | 1.045904691 | 1.479711107 | 0.013623764 |
|  | ACC | 1.757020346 | 1.091506739 | 2.828310981 | 0.020316599 |
|  | HNSC | 0.765508453 | 0.593841553 | 0.986800585 | 0.039157949 |
|  | KIRC | 0.573901714 | 0.466046439 | 0.706717507 | 1.71E-07 |
|  | OV | 1.293373683 | 1.104900949 | 1.51399588 | 0.001367814 |
| **EphA4** | UCEC | 1.293906894 | 1.060759469 | 1.578298471 | 0.011026911 |
|  | ACC | 1.710453242 | 1.113119777 | 2.628333762 | 0.014329324 |
|  | LGG | 1.201609167 | 1.026113268 | 1.407120087 | 0.022609259 |
|  | KIRP | 4.933795017 | 2.168043674 | 11.22778732 | 0.000142134 |
|  | CESC | 4.667022656 | 1.61360089 | 13.49844352 | 0.004469774 |
| **EphA5** | UVM | 4.263753229 | 1.188023977 | 15.30237768 | 0.026133479 |
|  | BRCA | 12.06663863 | 1.104486294 | 131.8294022 | 0.041208462 |
|  | READ | 2694654417 | 1.593451806 | 4.55688E+18 | 0.045183877 |
|  | UCEC | 3.298183247 | 1.801622607 | 6.037897553 | 0.000109696 |
| **EphA6** | READ | 122251.3971 | 54.28946751 | 275291042.2 | 0.002938349 |
|  | LIHC | 2.146253661 | 1.235169603 | 3.729370253 | 0.006744711 |
|  | STAD | 2.386551327 | 1.098136039 | 5.186631745 | 0.028067879 |
| EphA7 | KIRP | 0.73703088 | 0.590852865 | 0.919373587 | 0.006824842 |
|  | BLCA | 1.278196942 | 1.030108026 | 1.586035039 | 0.025787607 |
|  | KIRP | 1146.34698 | 19.47549918 | 67475.10738 | 0.000704081 |
|  | THCA | 344.9220024 | 6.142308589 | 19369.13231 | 0.00446648 |
| **EphA8** | STAD | 2.297374099 | 1.244662906 | 4.240447534 | 0.00781734 |
|  | GBM | 25.91495735 | 1.57407326 | 426.6542298 | 0.022762705 |
|  | UVM | 2.182726878 | 1.095533502 | 4.348836997 | 0.026460308 |
|  | LGG | 0.482541097 | 0.346858502 | 0.671299419 | 1.52E-05 |
|  | UCEC | 2.775267858 | 1.746871668 | 4.409088444 | 1.55E-05 |
|  | KIRP | 2.586214463 | 1.539750309 | 4.343889532 | 0.000329057 |
|  | KIRC | 1.77183631 | 1.264918494 | 2.481902133 | 0.000878767 |
| **EphA10** | PCPG | 2.446607959 | 1.24002505 | 4.827233532 | 0.009867759 |
|  | PAAD | 0.636066495 | 0.447313125 | 0.904468399 | 0.01176977 |
|  | KICH | 6.558494787 | 1.51312078 | 28.42724417 | 0.011954949 |
|  | LAML | 0.001393381 | 6.35E-06 | 0.305908161 | 0.016823223 |
|  | HNSC | 0.529829726 | 0.301285299 | 0.931739914 | 0.027423011 |
|  | THYM | 5.029589648 | 1.101632426 | 22.96298786 | 0.037078977 |

**Supplementary Figure 1.** We analyzed the effect of each EphA gene expression on the prognosis of specific cancers. This entailed an initial computation of survival data for EphA genes, followed by the exclusion of those lacking statistical significance in tumors, culminating in the extraction of the prognostic value of EphA family gene.
